# Indoor Trajectory Reconstruction of Walking, Jogging, and Running Activities Based on a Foot-Mounted Inertial Pedestrian Dead-Reckoning System

**DOI:** 10.3390/s20030651

**Published:** 2020-01-24

**Authors:** Jesus D. Ceron, Christine F. Martindale, Diego M. López, Felix Kluge, Bjoern M. Eskofier

**Affiliations:** 1 Telematics Engineering Research Group, Telematics Department, Universidad Del Cauca (Unicauca), Popayán 190002, Colombia; jesusceron@unicauca.edu.co; 2 Machine Learning and Data Analytics Lab, Computer Science Department, Friedrich-Alexander University Erlangen-Nürnberg (FAU), 91052 Erlangen, Germany; christine.f.martindale@fau.de (C.F.M.); felix.kluge@fau.de (F.K.)

**Keywords:** trajectory reconstruction, stride segmentation, dynamic time warping, pedestrian dead-reckoning

## Abstract

The evaluation of trajectory reconstruction of the human body obtained by foot-mounted Inertial Pedestrian Dead-Reckoning (IPDR) methods has usually been carried out in controlled environments, with very few participants and limited to walking. In this study, a pipeline for trajectory reconstruction using a foot-mounted IPDR system is proposed and evaluated in two large datasets containing activities that involve walking, jogging, and running, as well as movements such as side and backward strides, sitting, and standing. First, stride segmentation is addressed using a multi-subsequence Dynamic Time Warping method. Then, detection of Toe-Off and Mid-Stance is performed by using two new algorithms. Finally, stride length and orientation estimation are performed using a Zero Velocity Update algorithm empowered by a complementary Kalman filter. As a result, the Toe-Off detection algorithm reached an F-score between 90% and 100% for activities that do not involve stopping, and between 71% and 78% otherwise. Resulting return position errors were in the range of 0.5% to 8.8% for non-stopping activities and 8.8% to 27.4% otherwise. The proposed pipeline is able to reconstruct indoor trajectories of people performing activities that involve walking, jogging, running, side and backward walking, sitting, and standing.

## 1. Introduction

Indoor positioning systems (IPS) enable the provision of several location-based services such as home monitoring, rehabilitation, navigation for blind and visual impaired people, and finding and rescuing people/firefighters in emergencies. IPSs can be divided into two approaches: infrastructure-based and infrastructure-free [1,2]. Infrastructure-based IPS require the deployment of devices in the indoor environment to calculate the position of the person. Among the technologies used by this type of IPS are Wi-Fi [3], radio frequency identification (RFID) [4], Bluetooth [5], ultra-wide band (UWB) [6], infrared [7], and video cameras [4]. Infrastructure-free IPS do not need the deployment of devices and mainly use dead-reckoning algorithms. Those systems are called inertial pedestrian dead-reckoning (IPDR) because they use body movement information measured by inertial measurement units (IMU) to estimate a person’s position changes based on a previously estimated or known position [2]. The sum of these changes of position allows the reconstruction of the person’s trajectory [2]. An IMU usually consists of a triaxial accelerometer and gyroscope. Although some IMUs also incorporate a triaxial magnetometer, alterations of the magnetic field indoors make it unreliable for indoor positioning [8].

The advantages of IPDR systems over infrastructure-based systems are generally lower cost, data privacy, and ease of deployment. However, IPDR systems without correction suffer from severe drift, as person displacement is often calculated by integrating acceleration data from the accelerometer twice and integrating the rotational angle from the gyroscope. In consequence, intrinsic errors and IMU noise are raised to the third power, making a person’s trajectory reconstruction by direct integration without correction impractical [9,10,11].

The literature review done in this study is aimed at foot-mounted IMU IPDR systems that only use the accelerometer and/or gyroscope. Foot-mounted IPDRs, together with a zero velocity update (ZUPT) algorithm, have been the most widely and successful method used to mitigate the drift in trajectory reconstruction [9]. We use only the accelerometer and gyroscope because in indoor environments, different sources might produce alterations in the magnetic field that make the magnetometer readings unreliable for trajectory reconstruction [8]. Most of the foot-mounted IPDR systems that only use accelerometer and gyroscope data are based on trajectory reconstruction during normal walking. Natural movements like avoiding obstacles, sitting, swinging legs, stopping, or performing activities like jumping, jogging, or running have rarely been considered [9,10]. In consequence, the literature review is focused on the foot-mounted IPDR systems that have reconstructed the trajectory of walking, jogging, and/or running activities. Thus, only six studies met the inclusion criteria and are part of the literature review. The foot-mounted IPDR systems are usually evaluated in closed-loop trajectories by measuring the return position error (RPE). The RPE indicates the distance between the final position of the person obtained by the system and the actual physical final position of the person at the end of the trial [8].

Threshold-based and machine learning-based foot-mounted IPDR approaches have been proposed to deal with walking and running activities [12,13,14,15,16]. Li et al. [12,13] proposed a threshold-based stance-phase detector that consists of one footstep detector and two zero velocity detectors, one for walking and another for running. The evaluation of the system was done with one pedestrian who followed two closed-loop trajectories while walking and running. For the square-shape path (195.7 m), the RPE was 0.24% for walking and 0.42% for running. For the eight-shape path (292.1 m), the RPE was 0.2% for walking and 1.01% for running. An adaptive zero-velocity detector that selects an optimal threshold for zero-velocity detection depending on the movement (walking or running) of the person was proposed by Wagstaff et al. [15]. This system was evaluated by five people who walked and ran a distance of 130 m in an “L” shaped path. The RPE reported were 1% for walking and 3.24% for running.

Considering that zero-velocity detection using machine learning-based IPDR systems is free of threshold-tuning, Wagstaff et al. proposed a method for zero-velocity detection by using a long short-term memory neural network (LSTM) [16]. Five people walked and ran a 220-m “L” shaped path. The RPE in walking was 0.49% and running 0.93%. Similarly, Ren et al. proposed a zero-velocity detection algorithm based on HMM [14]. The system was evaluated by one person in an oval-shaped sports field of 422 m. The RPE when walking and running was 0.6% and 1.61%, respectively.

The described works have obtained very high precision in the trajectory reconstruction of walking and running. However, the systems were evaluated with very few participants, and the evaluated trajectories involved continuous walking and running activities. Currently, trajectory reconstruction methods in realistic scenarios—with several people, and considering walking, jogging, and running strides—are still missing.

Physical activity classification and gait event detection are key components of the trajectory reconstruction process using IPDR. Machine learning has played an important role in both topics. In [17] it is shown how different machine learning-based algorithms are able to classify different physical activities, including standing, sitting, walking, and running. Gait event detection has been performed by using several machine learning algorithms such as deep learning [18], hidden Markov models (HMM) [19,20] and neural networks [21,22].

The aim of the present work was to propose a pipeline for trajectory reconstruction using a foot-mounted IPDR system able to reconstruct the trajectories of activities that involve walking, jogging, and running strides as well as natural movements like stopping, standing, sitting, and side-walking.

This paper contributes to foot-mounted IPDR systems by (1) comprehensively evaluating the trajectory reconstruction of activities that involve walking, jogging, and running strides including the discrimination of natural activities such as stopping, sitting, and side-walking; and (2) evaluating two algorithms for Toe-off and Mid-Stance detection during walking, jogging, and running strides adapted from the ones proposed by Barth et al. [23].

The proposed pipeline is able to recognize walking, jogging and running strides and detect the Toe-off and Mid-Stance events in each of them. With this information, a foot-mounted IPDR system is able to reconstruct the person’s trajectory regardless of their gait speed. This allows the development of new ambient assisted living applications in which indoor tracking is a ground technology as well as the development of new applications for indoor sports.

## 2. Datasets

### 2.1. Unicauca Dataset

**Figure 1:**
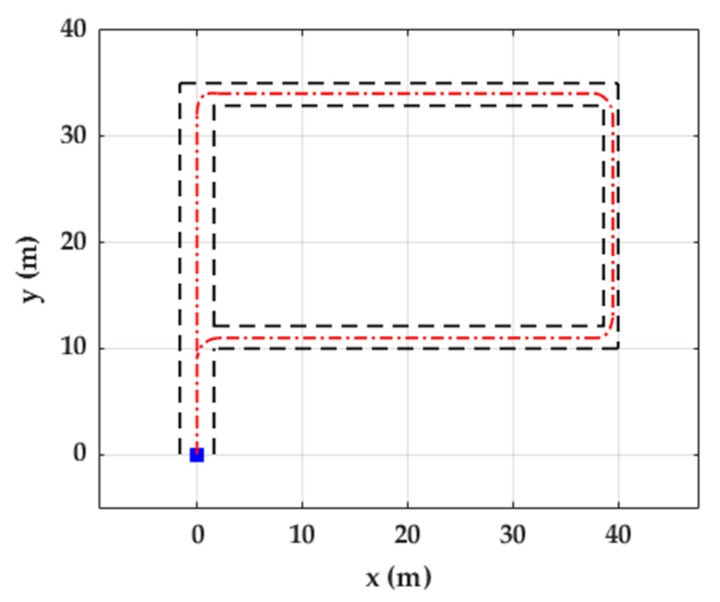
Illustration of the path used for walking, jogging and running in the Unicauca dataset. It is a “P” shaped path. The dotted red line represents the trajectory followed by one person, dotted black lines show outer edges (walls) of the path, and the blue square shows the start and end point of the trajectory.

**Figure 2:**
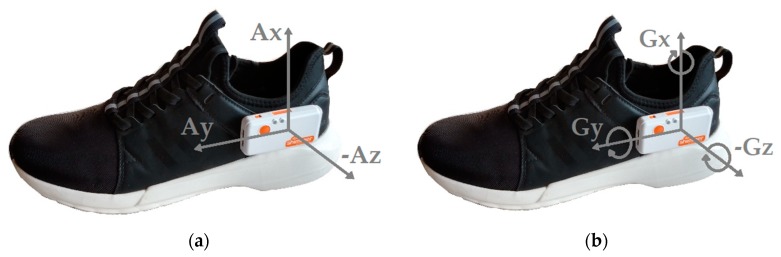
IMU sensor placement and axis alignment. (**a**) Accelerometer. (**b**) Gyroscope.

The objective of the Unicauca dataset was to evaluate the trajectory reconstruction of walking, jogging, and running in similar settings as the state-of-the-art methods, which are usually evaluated in close-loop trajectories and the activities performed by the participants include continuous walking, jogging, or running. This dataset was collected at the University of Cauca, Popayán, Colombia. Ten participants (mean age: 30 ± 3 years) walked, jogged, and ran a closed-loop P-shaped path of approximately 150 m (Figure 1) with an IMU attached to the lateral side of the left shoe with a Velcro strap (Figure 2).

The IMU was a Shimmer3 GSR+ (Shimmer Sensing, Dublin, Ireland). Acceleration (range: ±16 g) and angular velocity (range: ±2000 dps) data were collected at a frequency of 200 Hz. Accelerometer calibration consisted in leaving the sensor still for a few seconds lying on each of its 6 sides on a flat surface. For gyroscope calibration, the sensor is rotated around the three axes. At the beginning of each trial, the participant was asked to remain standing without moving the IMU for at least 10 s for gyroscope bias calculation.

### 2.2. FAU Dataset

**Figure 3:**
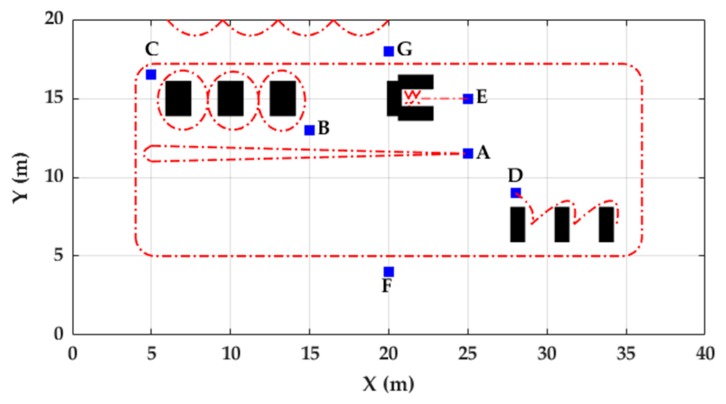
Map of the indoor environment used for collecting the FAU dataset. Blue squares represent chairs that denote start/end positions of activities. Black rectangles represent tables, and dotted red lines represent the possible trajectories followed by participants in each activity.

The FAU dataset is based on a previous study evaluating a method for smart labeling of cyclic activities [24] and is publicly available at www.activitynet.org. The dataset provides gait data in a relatively natural setting, and its protocol consisted in the execution of 12 different task-driven activities performed in random order for each participant. It includes data from 80 healthy participants with a mean age of 27 ± 6 years. Data were collected from 56 participants at the Friedrich-Alexander University Erlangen-Nürnberg (Germany) and from 24 participants at the University of Ljubljana (Slovenia). In this study, data collected at Slovenia from 20 of the 24 participants (mean age of 28 years) was used as training dataset [25] and data collected in Germany from the 56 participants were used as evaluation dataset. Only the data collected from the IMU worn on the left foot was used for trajectory reconstruction of ten activities (Table 1). Sensor placement and axis alignment are the same used in the Unicauca dataset (Figure 2). The acceleration (range: ±8 g) and angular velocity (range: ±2000 dps) were collected at a frequency of 200 Hz. The on-ground and off-ground phases of each stride are labeled. The accelerometer was calibrated using six static positions and the gyroscope was calibrated using a complete rotation about each of the three axes. Data were acquired in an indoor environment which including chairs and tables (Figure 3). Jogging was described to the participants as “if one would jog for exercise in the evening” and running as “if one is late for a bus”. These instructions were the same used in the Unicauca dataset.

## 3. Methods

**Figure 4:**
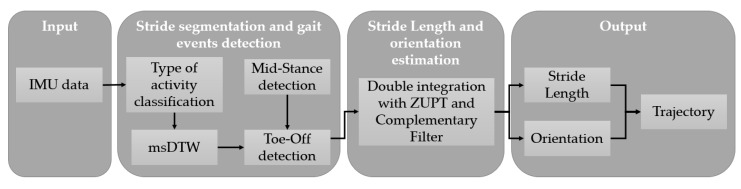
Pipeline for trajectory reconstruction for each activity.

A trajectory reconstruction pipeline was carried out separately for each activity of both datasets (Figure 4). This pipeline is based on previous work by Hannink et al. [26]. A type of activity classification step was included. Toe-Off and Mid-Stance algorithms were modified in order to deal with non-walking strides as well as a complementary filter added for stride length and orientation estimation.

### 3.1. Stride Segmentation

As shown by Zrenner et al., a threshold-based stride segmentation and a double integration with the ZUPT algorithm performed better than other approaches based on stride time, foot acceleration, and deep learning for calculating stride length in running using a foot-mounted IMU [27]. Thus, multi-dimensional subsequence dynamic time warping (msDTW) and a double integration with ZUPT were used as the stride segmentation and stride length and orientation estimation methods, respectively, in this study [23].

**Figure 5:**
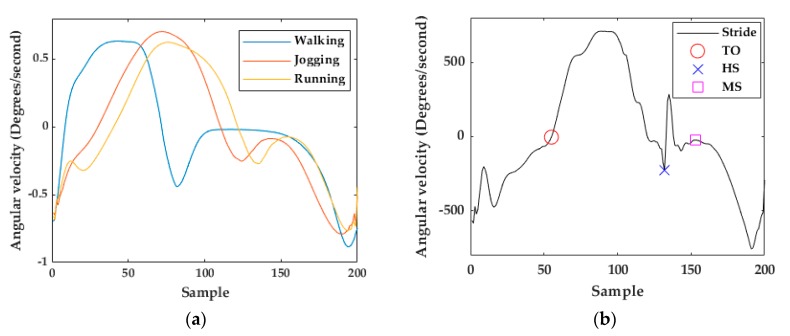
(**a**) Walking, jogging, and running templates (gyroscope z-axis). (**b**) Running stride example (gyroscope z-axis).

msDTW is used to find a subsequence of continuous signal sequences similar to a given reference pattern. In the context of stride segmentation, that pattern consists of a template of one stride. The stride start was set to the negative peak before the swing phase and stride end to the negative peak at the end of the stance phase (Figure 5a), according to the definition of stride given in [20]. Using that template, msDTW looks for similarities in a movement sequence. msDTW has been shown to be a robust method to segment strides from healthy, geriatric, and Parkinson’s patients using foot-mounted IMUs [28].

#### 3.1.1. Template Generation

A MatLab script was developed for template generation. It included two steps: interpolation and averaging. Interpolation consisted of taking each stride and interpolating it to a fixed duration of 200 samples. After interpolation, the template was obtained by averaging, sample by sample, all the strides. The templates for walking, jogging, and running were built using the 8724, 1688, and 1360 walking, jogging, and running strides, respectively, of the training dataset. Unlike other studies, which used only straight strides for building templates [23,28,29], the three templates were built with all the strides of the activities. Thus, both straight and non-straight strides were included in the templates.

The swing-phase starts when the foot leaves the ground (Toe-Off) and ends when the heel strikes the ground (Heel Strike). The portion of the gyroscope z-signal after Heel Strike (HS) describes the stance-phase. A Mid-Stance (MS) event is defined as the part of the stance-phase when the signal energy is zero [30].

#### 3.1.2. Classification of Walking, Jogging, and Running Activities

In order to automatically select the walking, jogging, or running template that will be used in the stride segmentation process, the machine learning algorithms included in the Matlab Classification Learner app were trained using the activities of the training dataset. A window size of 200 samples (1 s of data) and an overlap of 100 samples were used for feature extraction. The features extracted were velocity (by integrating accelerometer readings), angular velocity (by integrating gyroscope readings) and energy of accelerometer and gyroscope axes. The most frequent value in the result was chosen as the final classification. The evaluation was performed using ten-fold cross-validation. As a result, the highest accuracy (98.1%) was achieved by the SVM classifier with a polynomial kernel function of third-order.

#### 3.1.3. Multi-Subsequence Dynamic Time Warping Implementation

**Figure 6:**
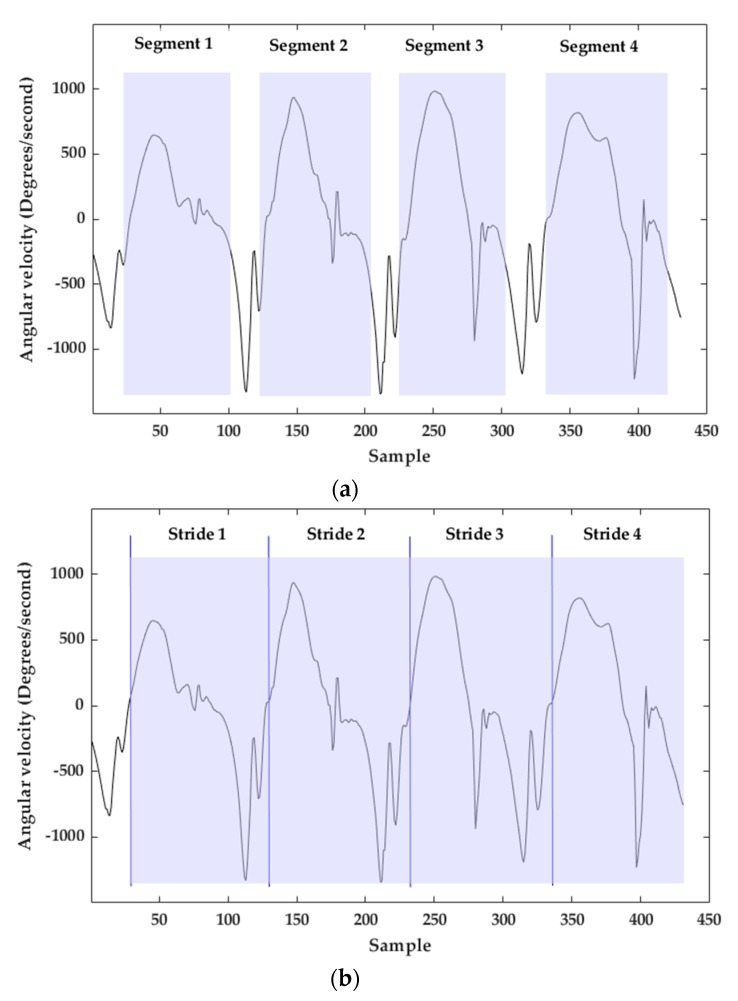
(**a**) Result of stride segmentation with msDTW. (**b**) Final stride segmentation with TO detection. Blue vertical lines depict TOs. Light blue rectangles are segmented strides.

The output of the stride segmentation based on msDTW is a set of segments [31]. Each segment describes a possible stride. One issue using these resulting segments for trajectory reconstruction is that often the end of a segment does not coincide with the start of the next segment even for consecutive strides (Figure 6a). The solution to this issue is based on the Toe-Off (TO) detection, which is described in the next section. Using the templates (Figure 5a), the first event detected in each stride is TO. For this reason, TO was defined as the beginning of a stride. For consecutive strides, the end of the stride corresponds with the beginning of the next stride (next TO), resulting in a stride segmentation without “holes” (Figure 6b).

**Figure 7:**
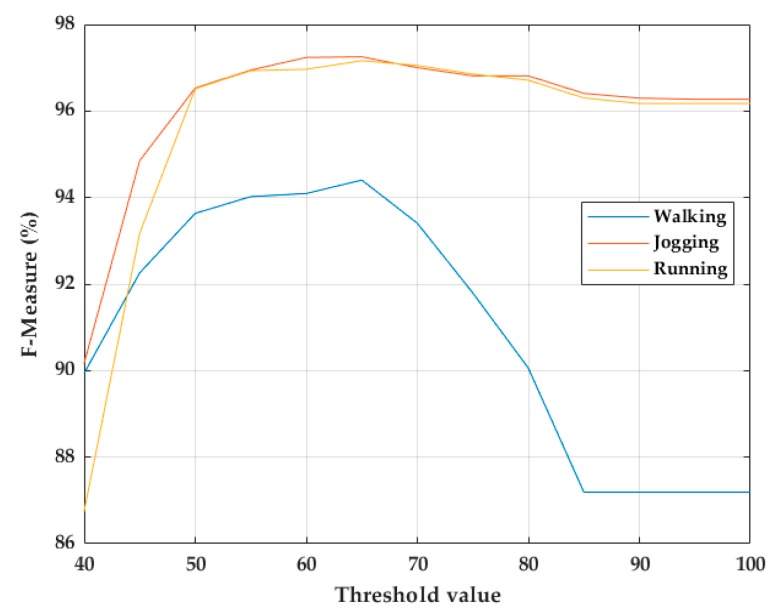
Threshold choice for stride segmentation of walking, jogging, and running strides using msDTW.

The precision and sensitivity of the stride segmentation using msDTW can be tuned using a threshold. The threshold needed to detect a stride indicates the similarity between that stride and the template used, that is, a large threshold indicates a large difference between the template and the segmented stride [23]. Therefore, with a very small threshold, the number of false negatives strides would increase, and a very large threshold would generate false positives strides. Thresholds from 0 to 100 in steps of 5 were tested on the training dataset. As a result, it was found that a fixed threshold of 65 maximizes the F-score of the stride segmentation in walking, jogging, and running activities (Figure 7).

### 3.2. Toe-Off and Mid-Stance Detection

The previous algorithms for TO and MS detection [31] were modified in order to improve detection accuracy in jogging and running. These modifications are described in this section. Both previous and proposed algorithms use the signal of the gyroscope *z*-axis for TO and MS detection.

#### 3.2.1. To Detection

**Figure 8:**
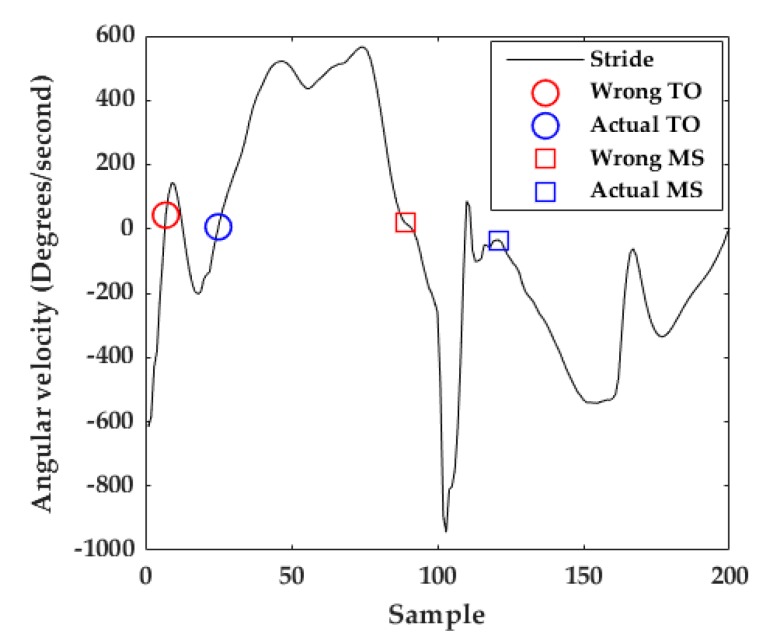
Example of TO and MS detection. The red circle and square show a wrong TO and MS detection, respectively, using the previous TO detection algorithm. The blue circle and square show an adequate TO and MS detection, respectively, using the proposed algorithms.

At TO, the gyroscope *z*-axis describes a zero-crossing because of the ankle joint changes from plantar flexion to a dorsal extension position in the sagittal plane [23]. The algorithm included in [31] for TO detection consists of detecting the first zero-crossing in the gyroscope *z*-axis. Due to the abrupt movements in jogging and running strides, in a few cases, a peak located at the beginning of the stride causes a zero crossing. This would lead to a wrong TO detection (red circle in Figure 8). Consequently, the adapted algorithm for TO detection (Algorithm 1) find the maximum peak of the signal and then find the nearest zero crossing before it (blue circle in Figure 8). After the detection of all the TOs that belong to the activity, all the portions corresponding from TO to TO are considered as strides (Figure 6b). Considering that the stride time of walking strides is around one second [24], if one TO to TO portion is greater than 2 s (400 samples), only the signal until 1.5 s was taken into account. This often happens because the participant is standing still or sitting.
**Algorithm 1: Toe-off (TO) detection algorithm.**1: *xMP ← getMaximumPeak(stride)* *2: xZC ← getZeroCrossings(stride(1 : xMP))* *3: TO ← getNearestZCtoMP(xZC, xMP)*

#### 3.2.2. Mid-Stance Detection

At Mid-Stance (MS) we define that the foot is entirely stationary on the ground [23,28] and its velocity is zero. The gyroscope *z*-signal is minimal at that moment. As the speed of movement increases from walking to running, the stance-phase time decreases (Figure 5a) making MS detection more difficult [10]. The previous algorithm for MS detection in walking strides consists of calculating the middle of the window with the lowest energy in the full stride’s gyroscope *z*-signal [23,28,31]. For jogging and running strides, the MS is often confused with other parts of the signal like the valley just before the HS or the peak before the next TO (red square in Figure 8).

The adaptation of the MS detection algorithm (Algorithm 2) consisted of (1) taking only the stride portion from HS to 80% of the stride—this portion was chosen taking into account that the stance-phase of walking strides is approximately the last 60% of the stride and for jogging and running strides it is approximately the last 40% of the stride [25]; (2) calculating the middle of the window with the lowest energy within that portion—to this end, a window size of 20 samples (100 ms) and a window overlap of 10 samples (Blue square in Figure 8) are used.
**Algorithm 2: Mid-Stance (MS) detection algorithm.**1: *windowSize ← 20* *2: overlap ← 10* *3: stride ← interpolateStrideTo200Samples(stride)* 4: *xMP ← getMaximumPeak(stride)* *5: stride ← stride(xMP : 160)* *6: xHS ← getMinimumPeak(stride)* *7: stride ← stride(xHS : end)* *8: MS getMinimumEnergy(stride, windowSize, overlap)*

#### 3.2.3. Stride Length and Orientation Estimation

The biggest challenge to adequately estimate stride length using IMU data is the significant bias derived from the use of IMUs, which leads to large drifts after the double-integration process. For that reason, the ZUPT method was used. Zero-velocity detection was done by evaluating a threshold on the magnitude of the gyroscope rate of turn of each measurement. If the measurement is less than a threshold of 0.6 dps, that measurement is considered as a zero-velocity measurement. It has been proved that this simple approach works properly in walking strides [11,30]. However, this approach does not work correctly in jogging and running strides due to the abrupt signal variations. The solution to this problem is the use of the MS detected previously. Taking into account that the average stance-phase time in running strides is around 100 ms (20 samples), it was empirically found that taking 5 samples to each side of the MS (which corresponds to 50 ms with the sampling frequency used) leads to better zero-velocity detection in jogging and running strides.

After zero-velocity detection, a complementary Kalman filter (CF) was used in order to model the error in velocity and position estimates using the ZUPTs as measurements (see Appendix A for details). When zero-velocity is detected, but the estimated velocity is different to zero, the CF adjusts the velocity and the corresponding displacement. The CF used in this work is based on the proposed work by Fischer et al. [11]. Three main parameters have to be set up for CF initialization: accelerometer and gyroscope noise ($\sigma_{a}$ and $\sigma_{w}$) and the ZUPT detection noise ($\sigma_{v}$). Accelerometer and gyroscope noise were set to equal value in both datasets ($\sigma_{a}=0.01\;m/s^{2}$ and $\sigma_{w}=0.01\;rad/s$). ZUPT detection noise depends on the velocity of the participant. That parameter was established by evaluating from $\sigma_{v}=0.001\;m/s$ to $\sigma_{v}=0.05\;m/s$ in steps of 0.001 m/s for each trajectory performed. The $\sigma_{v}$ chosen was the one that produced the least error in the final distance evaluated. The stride length and orientation estimation are obtained using the position increments in each MS event. Stride length, where $\nabla P_{k}$ is the position increment from stride k-1 to stride k, is calculated as follows:

(1) $$SL_{k}=\;\sqrt{\nabla P_{k}{\left(x\right)}^{2}+\;\nabla P_{k}{\left(y\right)}^{2}},$$

## 4. Results

### 4.1. Unicauca Dataset

#### 4.1.1. Classification of the Type of Activity

**Figure 9:**
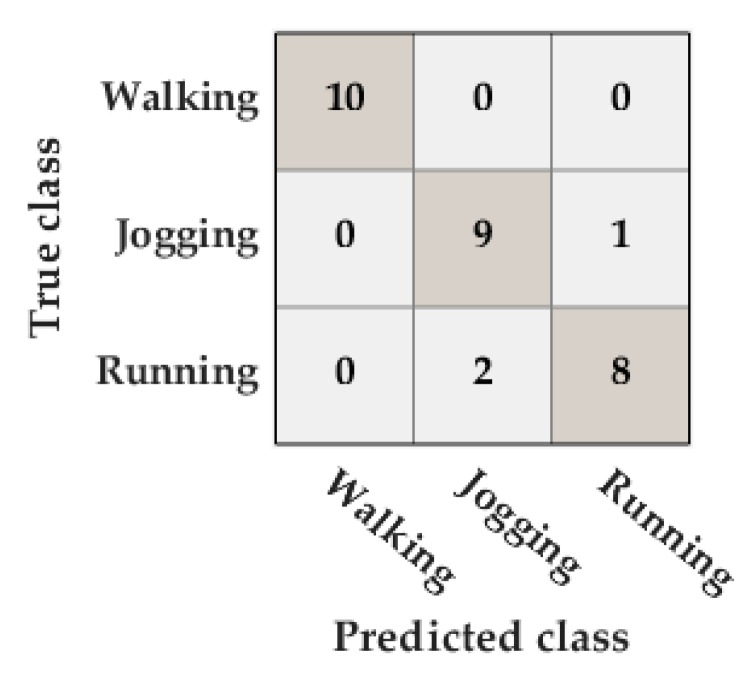
Confusion matrix of the classification of the type of activity in the Unicauca dataset.

The accuracy in the activity classification was 90%. There were only three misclassifications: two running activities were classified as jogging activities and one jogging activity was classified as a running activity (Figure 9).

#### 4.1.2. Toe-Off and Mid-Stance Detection

**Figure 10:**
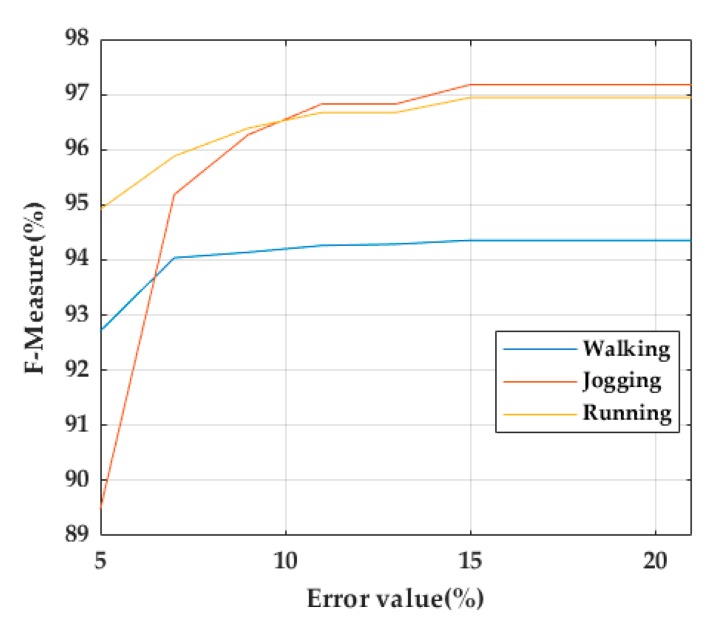
TO performance evaluation using error ranges from 5% to 21% in steps of 3%.

In this dataset, TO and MS were manually labeled. A TO/MS is considered as a true positive (TP) if it is located within 15% of the total number of samples of the stride to the right and left of the TO/MS ground truth. A false positive (FP) occurs when a TO/MS is detected outside this range. A false negative (FN) indicates that a TO/MS for a stride was not detected. Having in mind that 40% and 60% of the stride corresponds to the stance-phase of walking and running strides, respectively [25], the TO detection performance was evaluated in the training dataset using error ranges from 5% to 21% of the total stride in steps of 3% (Figure 10). As a result, 15% was chosen as an acceptable error range for TP calculation.

Results of the evaluation of the TO and MS detection using the previous and proposed algorithms are shown in Table 2 and Table 3, respectively.

A perfect F-score was obtained for TO and MS detection in walking strides. Very few mistakes occurred for jogging and running, but the F-score remains high.

#### 4.1.3. Trajectory Reconstruction

**Figure 11:**
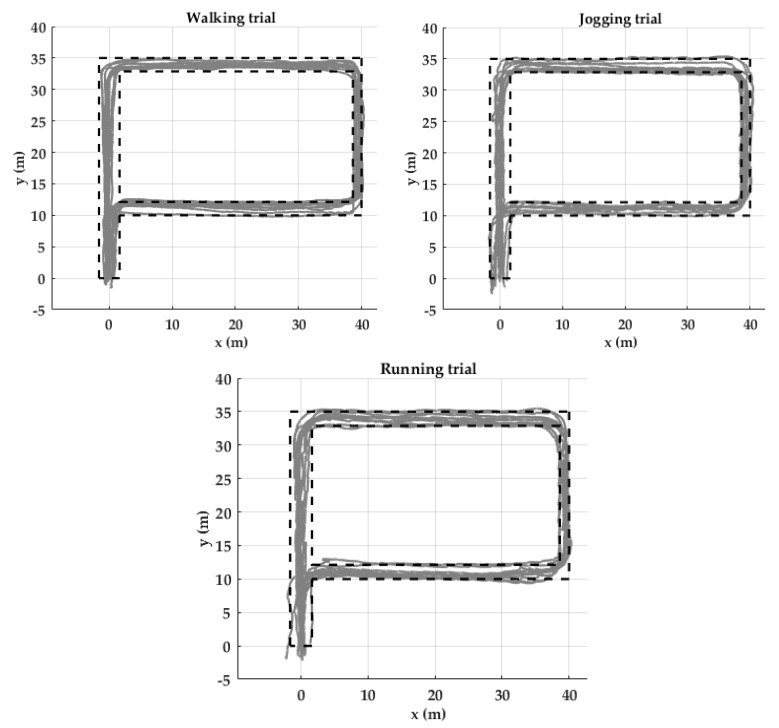
Trajectory reconstruction for the ten participants of the Unicauca dataset in a P shaped path. Black dotted lines show outer edges (walls) of the possible path. Gray lines are the trajectories reconstructed of the ten participants by using the proposed pipeline.

Two evaluation measures were used. (1) Return position error (RPE): the distance between the coordinates of the actual final point of the activity and the coordinates of the participant’s final stride of the corresponding activity. (2) Strides out of trajectory (SOT): All strides of the reconstructed trajectory should be within the boundaries of the corridors represented by black dotted lines (Figure 11). Otherwise, those strides will be counted as out of trajectory.

Higher velocity corresponds to more SOT and RPE. Although, on average, 5.7 % of the strides are out of trajectory in the running trial, the RPE remains less than 1.0% (Table 4). Trajectories of the three trials are mostly within the boundaries (Figure 11).

### 4.2. FAU Dataset

#### 4.2.1. Classification of the Type of Activity

**Figure 12:**
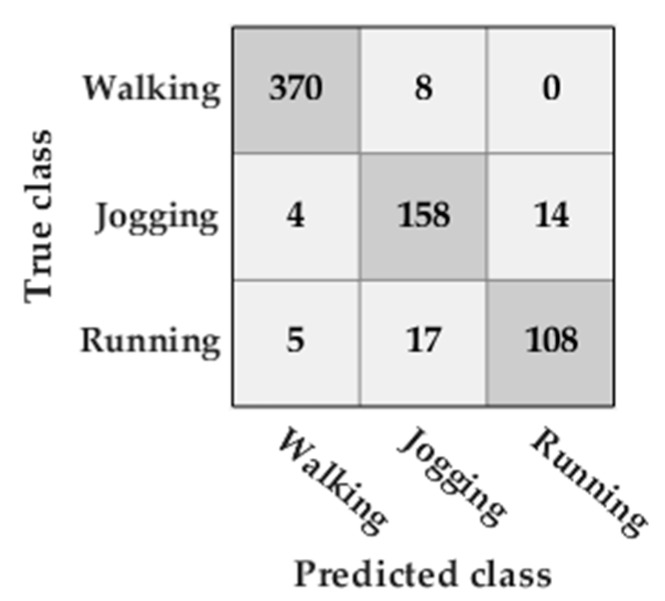
Confusion matrix of the classification of the type of activity classification in the FAU dataset activities.

The accuracy obtained by the SVM classifier was 93%. Most of the misclassifications occurred when classifying between running and jogging (Figure 12).

#### 4.2.2. Toe-Off Detection

The last sample of the on-ground phase of each stride was used as ground truth for the evaluation of the TO detection algorithm (Table 5). The same criteria used in the Unicauca dataset for TP, FP, and FN calculations were used. The evaluation was carried out on the data collected from the 56 participants at the Friedrich-Alexander University Erlangen-Nürnberg (Germany) of FAU dataset.

#### 4.2.3. Body Trajectory Reconstruction

**Figure 13:**
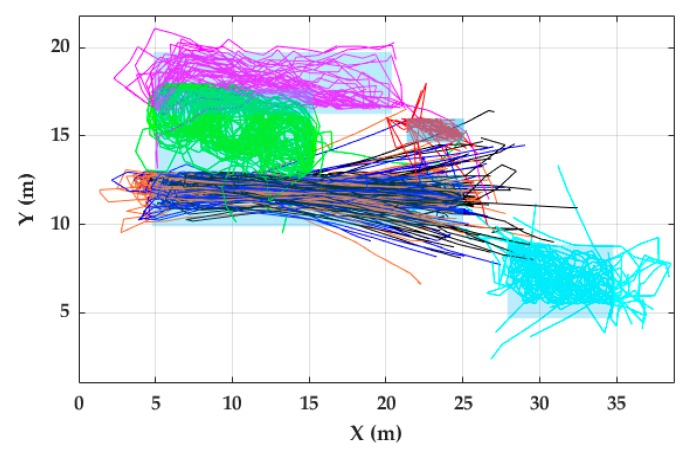
Trajectory reconstruction of non-circuit activities for all 56 participants of the FAU dataset. Black, blue and orange lines denote R-20, J-20, and W-20, respectively. Red, green, violet and light green lines represent W-Cards, W-Slalom, W-Posters, and W-Tables, respectively. Gray rectangles represent zones where all the strides related to certain activity should take place.

**Figure 14:**
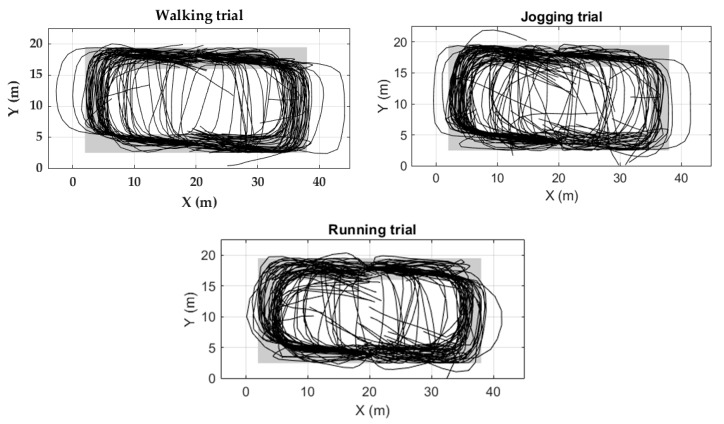
Trajectory reconstruction of circuit activities for all 56 participants of the FAU dataset. Black lines denote the trajectory follows by the participants. Gray zones represent the zone where all the strides should take place.

For RPE estimation in FAU dataset (Table 6), it is important to note that the start/end activity positions were defined by chairs in the indoor environment. For that reason, the actual positions where the participants started and finished the activities were not precisely the same as the chairs’ positions since participants began each activity near the corresponding chair and did not necessarily return to the exact point where they started the activity. Based on the videos of the data collection, participants started and finished the activities within a radius of 1.5 m around the chairs. Light blue and gray rectangles in Figure 13 and Figure 14, respectively, indicate the path where all the strides related to a certain activity should take place. If a stride is out of this path, it is considered as a Stride Out of Trajectory (SOT). A SOT can be caused by the accumulative error of stride lengths and angle calculation of previous strides. These zones were defined taking into account the coordinates of the chairs and tables and the boundaries of the indoor environment.

Most of the trajectories were inside the zones (Figure 13 and Figure 14). The trajectory reconstruction of activities W-20, J-20, and R-20 describes two straight trajectories, joined by a 180-degree turn. The trajectory reconstruction of W-Slalom allows sight of the area where the tables are located. The W-Posters activity includes non-straight strides, which are well described in the trajectory obtained. Regarding the circuit activities, although most of the strides are inside the activity zones, some trajectories lead towards the outer part of the activity zone. Others lead towards the internal part of the circuit (Figure 13).

## 5. Discussion

We have proposed a pipeline for indoor trajectory reconstruction of walking, jogging, and running activities. The proposed pipeline was evaluated with two datasets. The results showed that it is able to reconstruct a person’s trajectory regardless of their gait speed.

### 5.1. Classification of the Type of Activity

It was found that the classification model obtained with the SVM algorithm is able to classify the three types of activities performed: walking, jogging, and running. The classification between jogging and running is the one in which the classifier made more mistakes. This is possibly due to the jogging and running speeds of some participants being similar. The use of personal models to avoid this problem could be promising.

### 5.2. TO and MS Detection

Previous studies focused on the reconstruction of the trajectory during walking and running and do not show results of segmentation or detection of strides [18,19,20,21,22]. The two datasets used in this study allow TO evaluation. In the case of MS detection, ground truth information was not available in the FAU dataset. Therefore, it was not possible to evaluate MS detection in that dataset. However, a high F-score was obtained in the detection of MS in the Unicauca dataset.

While the F-score obtained for the proposed TO and MS detection algorithms is similar to that obtained for the previous algorithms for walking activities, the F-score achieved for the proposed TO and MS detection algorithms outperformed that achieved for the previous algorithms for all jogging and running activities. That suggests that the proposed algorithms can detect those gait events in walking, jogging, and running strides. The number of false positives (FP) was always higher than the number of false negatives (FN). This could indicate that the threshold used for stride segmentation with msDTW might have been overestimated, since stride segmentation using a large threshold implies that there is a large difference between the template used and the segmented strides, leading to the detection of FP strides. However, it was checked that by reducing that threshold, the number of FN increased, causing a decrease in the F-score. Threshold-free methods based on machine learning techniques such as those used by Ren [20] and Wagstaff [22] would make the stride segmentation process straightforward by avoiding setting any threshold.

The lowest F-scores are obtained for three walking activities: W-Posters, W-Tables, and W-Cards, which might be due to the fact that those activities involve non-stride movements such as stopping, sitting, lateral and backward steps. This could be because the signal generated for those foot movements is different from the walking/running templates. This could be accounted for by using templates generated by those specific movements, as previously demonstrated in [29], where specific templates were generated for each specific activity such as ascending and descending stairs. Unfortunately, the wide range of possible natural foot movements makes this alternative hard to implement. A hierarchical hidden Markov model (hHMM) approach has proved to be a robust method for stride segmentation of walking activities that include non-stride movements in Parkinson’s patients [14] and for stride segmentation of jogging activities [15]. Furthermore, hHMM is a threshold-free approach, therefore it should be explored in order to improve the results obtained for the walking activities that include non-stride movements such as W-Posters, W-Tables, and W-Cards, as well as for stride segmentation of jogging and running activities.

### 5.3. Trajectory Reconstruction

Usually, the foot-mounted IPDR systems have been evaluated in closed-loop trajectories and by measuring the Return Position Error (RPE) [18,19,20,21,22]. The purpose of the Unicauca dataset was, therefore, to provide a starting point to allow a fair comparison with the state-of-the-art papers.

Sometimes the RPE is small, although the reconstructed trajectory does not fit the actual trajectory performed by the person. That is why we proposed the number of strides out of the trajectory as an additional evaluation metric. The RPEs obtained with the pipeline proposed in this paper for the three trials collected in the Unicauca dataset are less than 1%. The results obtained by the works described in the literature review section are also lower than 1%.

As a result of the better detection of TO and MS obtained by using the algorithms proposed in this study, there is also a better trajectory reconstruction since there were fewer strides out of trajectory (SOT) and shorter RPE for jogging and running activities. This demonstrates two things. The first is the importance of performing a correct detection of TO and MS for trajectory reconstruction. The second is that if the complementary filter does not have precise data to perform the ZVUs, it is not capable of modeling errors in speed on its own, even if its parameters were tuned. It has also been demonstrated that by properly detecting TO and MS, the complementary filter is capable of modeling errors in walking, jogging, and running strides.

RPE obtained for trajectories in the FAU dataset are higher than for the Unicauca dataset. It is important to highlight two limitations that the FAU dataset has for trajectory reconstruction. Firstly, the position of the participants at the beginning and end of the activities is not exactly the same. When analyzing the videos of the FAU dataset collection, it was concluded that these positions vary approximately in a radius of one and a half meters, taking as reference the chairs that indicated the start and end of the activities. Therefore, the RPEs calculated have an error of ±1.5 m. This fact should be taken into account for the preparation of the protocol for the collection of a future dataset. Secondly, it was not possible to subtract the gyroscope bias in all activities performed in the FAU dataset, because the activities were performed continuously. A prerequisite for bias computation is that the person stands still for a few seconds for the calculation of the mean of the gyroscope readings and then subtracting it from the entire movement sequence.

The number of strides out of trajectory is directly related to the RPE obtained; the more strides out of the acceptable path range, the higher the RPE. When observing the trajectory reconstruction of the activities W-20, J-20, R-20, and W-Circuit, J-Circuit, R-Circuit, it appears that the difficulty in trajectory reconstruction increases with stride velocity (from walking to jogging and running). This also occurred in the five papers described in the literature review section [18,19,20,21,22]. In those papers, the evaluation was performed with very few people. From our study, we can confirm that there is still a gap in trajectory reconstruction using foot-mounted IPDR systems of jogging/running activities regarding the trajectory reconstruction of walking activities.

The RPE of the trajectory reconstruction of W-Cards, W-Tables, and W-Posters activities are particularly high, due to the bad detection of TOs. These activities should be treated with special care in future works since they describe movements of daily living activities that happen frequently.

The trajectories obtained have a very well-defined shape and could be used for mapping an indoor environment.

One important recommendation for future work in the field of trajectory reconstruction using IPDR systems is that the datasets collected for evaluation are labeled at activity and stride/step levels, as the FAU dataset used in this paper. Additionally, the participants of the data collection process must start and end precisely at the indicated coordinates.

## 6. Conclusions

In this paper, we have proposed and evaluated a pipeline for trajectory reconstruction of walking, jogging, and running activities using a foot-mounted inertial pedestrian dead-reckoning system. The dynamic time warping method was adapted within this paper to segment walking, jogging, and running strides. Stride length and orientation estimation were performed using a zero velocity update algorithm adapted for walking, jogging, and running strides and empowered by a complementary Kalman filter.

The presented results showed that the proposed pipeline provides good trajectory estimations during walking, jogging, and running. TO detection algorithm reached an F-score between 92% and 100% for activities that do not involve stopping, and between 67% and 70% otherwise. Resulting return distance errors were in the range of 0.51% to 8.67% for non-stopping activities and 8.79% to 27.36% otherwise.

To the best of the authors’ knowledge, this is the most comprehensive evaluation of a foot-mounted IPDR system regarding the type and number of activities and quantity of people included in the datasets and can serve as a baseline for the comparison of future systems. Future work will be focused on using hidden Markov models in order to improve stride segmentation and fusing symbolic location from an RSSI signal to update the indoor localization when possible.

## Figures and Tables

**Table 1:** Activity descriptions and abbreviations, shown with their relevant start and end points as labeled in Figure 3 as well as approximated distances.

Activity	Description	Start/End Position	Approximated Distance (m)
W-Slalom	Walk slalom through 3 tables	B→B	31
W-Posters	Sign name on 5 posters on the wall	C→G	21
W-Tables	Perform task at 3 different tables while sitting	D→D	20
W-Cards	Perform task on a table while standing	E→E	6
W, J, R-20	Walk, jog, run 2 times 20 m	A→A	40
W, J, R-Circuit	Walk, jog and run half a circuit each	F, G→G,F	43

**Table 2:** Averaged results of TO and MS detection for the 10 participants in the Unicauca dataset using the previous TO and MS detection algorithms.

	Toe-Off					Mid-Stance				
Activity	TO GT	TP	FP	FN	F-Score (%)	MS GT	TP	FP	FN	F-Score (%)
Walking	105.5	105.4	0.1	0.1	99.9	104.5	104.4	0.1	0.1	99.9
Jogging	75.4	39.4	37.1	36.2	51.5	74.4	41.2	34.2	33.3	54.9
Running	59.6	21.7	37.5	37.1	36.4	58.6	25.8	31.5	30.7	45.3

TO GT: ground truth TO rate. MS GT: ground truth MS rate. TP: true-positive rate. FP: false-positive rate. FN: false-negative rate.

**Table 3:** Averaged results of TO and MS detection for the 10 participants in the Unicauca dataset using the proposed TO and MS detection algorithms.

	Toe-Off					Mid-Stance				
Activity	TO GT	TP	FP	FN	F-Score (%)	MS GT	TP	FP	FN	F-Score (%)
Walking	105.5	105.5	0	0	100	104.5	104.5	0	0	100
Jogging	75.4	75.2	0.1	0.2	99.8	74.4	74.4	0	0	100
Running	59.6	59.3	0.3	0.2	99.7	58.6	58.5	0.1	0.1	99.8

TO GT: ground truth TO rate. MS GT: ground truth MS rate. TP: true-positive rate. FP: false-positive rate. FN: false-negative rate.

**Table 4:** Average results of trajectory reconstruction for each type of activity performed by the 10 participants using the previous and the proposed TO and MS detection algorithms.

Activity	SOT				RPE			
	[31]		New A		[31]		New A	
	#	%	#	%	meters	%	meters	%
Walking	1.7	1.6	1.7	1.6	0.8	0.5	0.8	0.5
Jogging	6.6	8.6	2.9	3.8	2.2	1.4	1.4	0.9
Running	5.3	9.2	3.3	5.7	2.6	1.6	1.4	0.9

SOT: strides out of trajectory, RPE: return position error, [31]: previous TO and MS detection algorithms, New A: proposed TO and MS detection algorithms.

**Table 5:** Average results of TO detection for each type of activity performed by the 56 participants in the FAU dataset using the previous and the proposed TO detection algorithms.

Activity	TO	TP		FP		FN		F-Score (%)	
		[31]	New A.	[31]	New A.	[31]	New A.	[31]	New A.
W-Slalom	21.5	21.2	21.2	0.8	0.8	0.4	0.3	97	97
W-Posters	13.0	10.6	10.6	3.6	3.5	2.3	2.3	77	78
W-Tables	11.9	9.5	9.5	3.7	3.7	2.4	2.4	75	75
W-Cards	4.33	3.7	3.7	1.6	1.6	2.4	0.6	71	71
W-20	28.4	28.2	28.2	1.3	1.0	0.4	0.2	99	98
J-20	22.3	13.7	21.6	9.7	1.1	8.6	0.7	56	96
R-20	18.4	8.1	17.0	12.3	2.1	10.6	1.3	48	90
W-Circuit	28.2	27.6	27.7	0.7	0.7	0.4	0.3	98	98
J-Circuit	21.9	11.8	21.3	10.8	0.7	10	0.5	49	97
R-Circuit	17.7	7.6	17.3	10.7	0.8	10.2	0.4	40	96

TO: toe-off rate, TP: true positives rate, FP: false positives rate, FN: false negatives rate, [31]: previous TO and MS detection algorithms, New A: proposed TO and MS detection algorithms.

**Table 6:** Averaged results of trajectory reconstruction of activities performed by the 56 participants in the FAU dataset using the previous and the proposed TO and MS detection algorithms.

Activity	Activity distance	SOT				RPE			
		[31]		New A.		[31]		New A.	
	meters	#	%	#	%	meters	%	meters	%
W-Slalom	31	1.1	5.2	1.1	5.2	1.7	5.5	1.7	5.5
W-Posters	21	1.0	8.0	1.0	8.0	1.9	9.0	1.8	8.8
W-Tables	20	3.1	25.9	3.1	25.9	2.8	14.1	2.8	14.1
W-Cards	6	1.3	30.5	1.3	30.5	1.6	27.4	1.6	27.4
W-20	40	3.7	13.1	3.7	13.1	1.7	4.2	1.7	4.2
J-20	40	7.5	34.2	3.9	17.9	5.5	14.2	2.0	5.1
R-20	40	4.1	22.5	3.1	17.0	5.2	13.9	2.5	6.0
W-Circuit	43	4.1	14.4	4.1	14.4	2.9	6.7	3.0	6.7
J-Circuit	43	6.4	30.2	4.5	20.4	14.5	33.7	3.6	8.8
R-Circuit	43	5.1	29.9	3.9	22.4	16.2	37.7	3.7	8.7

SOT: strides out of trajectory, RPE: return position error, [31]: previous TO and MS detection algorithms, New A: proposed TO and MS detection algorithms.

## Appendix A. Complementary Filter

The initialization of the Complementary Filter (CF) implies to establish a series of matrices. First, the state of the CF includes the errors in orientation, position, and velocity. (A1) shows the state in an array representation. Each array element is a 1 × 3 array containing the errors in the three-axis.

(A1) $$E=\left[E_{o}\;E_{p}\;E_{v}\right]$$

The error covariance matrix accumulates the error in orientation, position, and velocity produced in each sample k:

(A2) $$P_{k}=[0_{9x9}]$$

The state transition function is a matrix that is multiplied with the previous state to get the next state, as shown in (A7). ‘S’ is the Skew-symmetric cross-product operator matrix formed from the n-frame accelerations and is the time step equals to 0.005 s, which results from dividing 1 s between the IMU data collection frequency (200Hz).

(A3) $$F_{k}=\;\begin{array}{ccc} I_{3X3} & 0_{3x3} & 0_{3x3}\\ 0_{3x3} & I_{3X3} & I_{3X3}∆t\\ -S∆t & 0_{3x3} & I_{3X3} \end{array}$$

The process noise covariance matrix is calculated for each sample by multiplying the accelerometer and gyroscope noise by:

(A4) $$Q_{k}=\;\left[\left(\sigma_{w_{x}}\;\sigma_{w_{y}}\;\sigma_{w_{z}}\;0\;0\;0\;\sigma_{a_{x}}\;\sigma_{a_{y}}\;\sigma_{a_{z}}\right)∆t\right]$$

The uncertainty in velocity during each ZUPT is represented using the measurement noise covariance matrix (A5). It is a diagonal matrix because no correlation in velocity is supposed to exist between axes.

(A5) $$R=\;\begin{array}{ccc} \sigma_{v_{x}}^{2} & 0 & 0\\ 0 & \sigma_{v_{y}}^{2} & 0\\ 0 & 0 & \sigma_{v_{z}}^{2} \end{array}$$

The measurement function matrix is used to move from the state variables space to the measurement variables states. In this implementation, the measurements are the ZUPTs that is when velocity is supposed to be zero. That way, the measurement function has to contain an identity matrix in the position of the velocity error state as follows:

(A6) $$H_{k}=\;\left[\left(0_{3x3}\;0_{3x3}\;I_{3x3}\right)\right]$$

Before running the CF, the gyroscope bias has to be removed. Gyroscope bias is obtained by calculating the mean of the gyroscope readings while IMU is not moving just before the beginning of the activity. The resulting value is subtracted to all gyroscope signals.

After gyroscope bias subtraction, the CF is executed. It has two phases: Prediction and update. In the prediction phase, the error covariance matrix ($P_{k}$) is propagated using (A7):

(A7) $$P_{k}=\;F_{k}P_{k-1}F_{k}^{T}+Q_{k}$$

Only when a sample k is a ZUPT, the Update phase comes into play. In this case, the Kalman gain is calculated with (A8), and with that gain, the error is obtained using (A9).

(A8) $$K_{k}=\;P_{k}H^{T}{\left(HP_{k}H_{T}+R\right)}^{-1}$$

(A9) $$E=\left[E_{o}\;E_{p}\;E_{v}\right]=\;K_{k}V_{k}$$

Finally, the velocity and position estimates are corrected as well as *P_k_*:

(A10) $$V_{k}=\;V_{k}-\;E_{v}$$

(A11) $$Pos_{k}=\;Pos_{k}-\;E_{p}$$

(A12) $$P_{k}=\left(I_{9x9}-K_{k}H\right)P_{k}$$
